# Determinants of uptake of hepatitis B testing and healthcare access by migrant Chinese in the England: a qualitative study

**DOI:** 10.1186/s12889-017-4796-4

**Published:** 2017-09-26

**Authors:** Andrew Chee Keng Lee, Alicia Vedio, Eva Zhi Hong Liu, Jason Horsley, Amrita Jesurasa, Sarah Salway

**Affiliations:** 1 Section of Public Health, the School of Health and Related Research, The University of Sheffield, Regent Court, Regent Street, Sheffield, S1 4DA UK; 2 Department of Infection and Tropical Medicine, Sheffield Teaching Hospitals NHS Foundation Trust, Sheffield, UK; 3 Public Health Department, Sheffield City Council, Sheffield, UK

**Keywords:** Hepatitis B, Chinese, Migrants, Healthcare access, Ethnicity

## Abstract

**Background:**

Global migration from hepatitis B endemic countries poses a significant public health challenge in receiving low-prevalence countries. In the UK, Chinese migrants are a high risk group for hepatitis B. However, they are an underserved population that infrequently accesses healthcare. This study sought to increase understanding of the determinants of hepatitis B testing and healthcare access among migrants of Chinese ethnicity living in England.

**Methods:**

We sought to obtain and integrate insights from different key stakeholders in the system. We conducted six focus group discussions and 20 in-depth interviews with community members and patients identifying themselves as ‘Chinese’, and interviewed 21 clinicians and nine health service commissioners. Data were thematically analysed and findings were corroborated through two validation workshops.

**Results:**

Three thematic categories emerged: knowledge and awareness, visibility of the disease, and health service issues. Low disease knowledge and awareness levels among community members contributed to erroneous personal risk perception and suboptimal engagement with services. Limited clinician knowledge led to missed opportunities to test and inaccurate assessments of infection risks in Chinese patients. There was little social discourse and considerable stigma linked to the disease among some sub-sections of the Chinese population. A lack of visibility of the issue and the population within the health system meant that these health needs were not prioritised by clinicians or commissioners. Service accessibility was also affected by the lack of language support. Greater use of community outreach, consultation aids, ‘cultural competency’ training, and locally adapted testing protocols may help.

**Conclusions:**

Hepatitis B among migrants of Chinese ethnicity in England can be characterised as an invisible disease in an invisible population. Multi-modal solutions are needed to tackle barriers within this population and the health system.

**Electronic supplementary material:**

The online version of this article (10.1186/s12889-017-4796-4) contains supplementary material, which is available to authorized users.

## Background

There are an estimated 326,000 persons chronically infected with hepatitis B virus (HBV) in the UK [1]. In low prevalence countries such as the UK, chronic infection, predominantly acquired abroad, accounts for much of the burden of hepatitis B [2]. Population groups who are more likely to be infected include migrants from high prevalence countries such as China and other Southeast Asian countries (including Hong Kong, Vietnam and Malaysia among others). Though China has recently been classified as having a high intermediate prevalence, (due to a sharp decrease in prevalence in children following the implementation of universal immunization) [3], the disease prevalence is up to twenty times higher among individuals self-identifying as ‘Chinese’ in the UK than in the British general population [2, 4, 5]. Migration-related chronic HBV infection is emerging as a significant public health issue in many western countries, increasing both the burden of disease as well as risks of disease transmission locally [6].

Prior research suggests that high risk ethnic minority groups are often underserved with low access to testing and healthcare by these groups previously reported in the UK [6, 7] and elsewhere, particularly the US and Canada [8–11]. Initiatives to boost the uptake of HBV testing in migrant Chinese populations have been tried, such as the *Jade Ribbon* campaign in San Francisco and *BeFreeNYC* in New York, with variable results [8, 9, 11]. Most of these initiatives have focused on ‘liver cancer prevention’ and sought to address perceived deficits in knowledge and disease awareness in these communities [12, 13]. Several studies identified obstacles to testing such as financial barriers (e.g. user fees or lack of health insurance), language barriers, and lack of familiarity with the host country’s health system [14, 15]. Other studies have found that opportunities for testing are often missed due to low levels of knowledge and disease awareness among clinicians [15–18].

The roles of health service planners and health systems are also rarely studied. In the UK, hepatitis B-related healthcare is provided through a range of different agencies – infectious disease screening is usually provided opportunistically through primary healthcare and community services, whilst treatment and long-term disease monitoring is delivered through specialist hospital-based services. These services are commissioned and funded separately by different agencies such as the local authorities, local clinical commissioning groups and the National Health Service at the regional or national level. Whilst there is a universal infectious diseases screening programme in pregnancy, the UK has adopted a policy of selective immunisation of newborns at highest risk of infection. It has been recognised that this immunisation policy poorly protects existing or new migrants with chronic HBV infection from high prevalence countries [1].

With increasing numbers of Chinese migrants globally [19, 20], health issues linked to hepatitis B present new challenges to health systems in receiving countries. However, outside North America, there is a dearth of studies about hepatitis B in migrant Chinese populations. This paper reports on a multi-perspective qualitative study in the UK that explores how migrant Chinese populations, clinicians and commissioners understand and experience this disease, and how these understandings and experiences impact on hepatitis B testing and healthcare access.

## Methods

### Study aim and design

The overall study aim was to identify barriers and enablers of hepatitis B testing and healthcare access by migrants of Chinese ethnicity living in England. These processes were explored sequentially through a series of qualitative investigations with community members and patients self-identifying as ‘Chinese’, healthcare practitioners, as well as managers and clinicians responsible for the planning and strategic purchasing of health services (referred to as ‘commissioners’). This approach was adopted as the uptake of HBV testing and healthcare access was thought likely to be influenced by complex interactions between various stakeholders, the health system and the wider health policy arena [21]. This multi-perspective approach, a unique strength of this study, sought to integrate and contextualise the knowledge and behaviours of migrant Chinese individuals, clinicians and commissioners within the backdrop of current health policy, organisation and delivery.

### Setting

This study was conducted between September 2013 and September 2015 in South Yorkshire, England. We defined our study population broadly to include any individual who self-identified as Chinese. This includes not only migrants born in China, but also those originating from other parts of East and Southeast Asia, such as Malaysia, Taiwan, and Vietnam, where there are established Chinese communities. They include different migration waves (including place of origin, time of migration) as well as different generations of migrants (e.g. children of first generation migrants). In addition, the Chinese communities in South Yorkshire are also geographically dispersed in the region. As such, the label ‘Chinese’ covers a heterogeneous group of individuals with diverse linguistic and cultural heritage and varied socioeconomic profiles.

### Community-level data collection

Six focus group discussions were conducted involving 31 Chinese adults resident in South Yorkshire recruited through community groups, local venues and via snowballing. The focus groups were organised around natural groupings to facilitate participation. They included mothers with young children, elderly residents, university students, parents of children attending a Chinese school, and a Chinese community centre group. We used a case vignette developed from initial ethnographic field work and a topic guide to facilitate discussions (see Additional file 1). A multi-lingual researcher facilitated three focus group discussions in Mandarin and three in Cantonese.

Subsequently, 20 in-depth face-to-face individual interviews were conducted with new participants recruited using the same recruitment channels as for the focus groups. Interviewees included 13 who reported having HBV infection, three relatives of individuals who had HBV infection, and four community workers. Interviews explored individual narratives and patient journeys. Preliminary findings from the focus group discussions informed the interviews and enabled some issues to be explored in greater depth, such as stigma and knowledge sources. Written informed consent was sought from all participants, and participants received reimbursements of their travel expenses for their involvement in the study where required. The interview guide used for the community interviews are provided in Additional file 2.

### Health service-level data collection

Individual, semi-structured interviews with clinicians were conducted to explore behaviour and attitudes towards hepatitis B testing and referral, as well as their personal experiences of dealing with hepatitis B and patients identified as ‘Chinese’. We recruited clinicians who were involved in hepatitis B care or provided healthcare to Chinese patients through purposive, convenience and snowballing sampling in order to obtain a range of views. We interviewed 21 clinicians drawn from five localities from different settings (hospitals, primary care and the community) and from different specialities (primary care, infectious diseases, sexual health, nephrology, rheumatology and midwifery services). Interviews were guided by an interview schedule (see Additional file 3) which drew on our initial literature review [22] and early findings from the community study.

### Commissioning-level data collection

We also purposively sought and interviewed clinicians and managers involved in commissioning or managing hepatitis B services or health services for ethnic minority groups. Potential participants were identified with the help of our expert advisory group, and existing links with various commissioning organisations. Snowballing was used to identify other key individuals who held relevant roles. Nine participants were recruited that included directors of public health, consultants in public health and communicable disease control, and senior commissioning managers working across a range of different commissioning organisations at local, regional and national levels. Individual interviews were conducted face-to-face using an interview schedule of open-ended questions (see Additional file 4) to explore participants’ awareness and knowledge of the disease, attitudes, perception of risk in the Chinese population, as well as perceived individual and system obstacles to the commissioning and delivery of relevant services.

Following written informed consent, focus group discussions and individual interviews were conducted in mutually convenient venues either in community venues, workplace or university setting, and lasted between 40 min and two hours. The interviews and discussions were audio recorded with the participants’ consent and carried out on the condition of participant anonymity.

### Data management, analysis and integration

Audio recordings were transcribed verbatim and subsequently translated where necessary for further analysis. For the community study, a set of thematic codes was developed drawing on our literature review [22] and initial ethnographic fieldwork. The coding scheme was iteratively developed by reading and re-reading the transcripts and through research team discussions. Once agreed, the thematic codes were applied systematically across the group discussion and individual interview transcripts. For the subsequent studies of clinicians and commissioners, deductive and inductive interpretive approaches were used to identify key themes. Descriptive coding and process coding were used to summarize passages of data and thematic content analysis was applied to organise and consolidate relevant themes [23].

Emergent themes from the different studies were integrated through a series of team workshops to identify key factors operating at individual, family, community and health service level that shape access, experience and outcomes of healthcare. We looked for matching themes and links between emergent themes, explored discordant themes and sought to understand reasons that could explain the discordance. Disagreements were discussed until consensus was reached. Complementary themes were grouped and re-grouped into higher themes and are presented in the following section.

Two validation workshops were conducted in Sheffield and London with stakeholders from across the country. There was representation from Public Health England, local authorities, clinical networks, primary care and Chinese community organisations. Participants included hospital physicians, general practitioners, commissioners, consultants in public health and communicable disease control, nurses, immunisation leads and community representatives. Stakeholders were identified with the help of our Expert Advisory Group, and purposively selected in order to have a breadth of perspectives. The workshops were used to assess the generalisability of the findings to other parts of the country.

## Results

A summary of participants’ characteristics is provided in Tables 1 and 2. Of the 51 community participants, only one turned out to have been born in the UK. This individual was retained in the study as it was judged not appropriate to exclude this individual from the focus group discussion that they were part of.

**Table 1:** Community participants’ characteristics

	Focus group participants (*n* = 31)	In-depth interviewees (*n* = 20)
Age-group (years)		
18–34	7	2
35–44	15	6
45–54	3	2
55–64	3	2
65–74	2	7
75+	1	1
Gender		
Female	27	14
Male	4	6
Country of birth		
China	15	15
Taiwan	5	0
Hong Kong	4	5
Vietnam	4	0
Malaysia	1	0
Brunei	1	0
UK	1	0
Years lived in the UK		
0–4	6	1
5–9	6	3
10–19	10	10
20+	9	6
Educational level		
Primary	2	2
Secondary	6	10
College/vocational school	5	0
Graduate	7	2
Postgraduate	11	5
Other	0	1
Current employment status		
Employed	11	5
Self-employed	2	2
Unemployed	1	3
Student	4	1
Homemaker	10	2
Retired	3	7
Annual household income		
< £25,000	9	7
£25,000–£49,000	11	4
£50,000+	2	0
Don’t know	9	9
Marital status		
Married	24	10
Single, never married	5	1
Divorced/separated	1	6
Widowed	1	3
Household size		
1	3	3
2–3	11	9
4	12	4
5+	5	3

**Table 2:** Characteristics of clinical participants

Setting	Practitioner type	Number interviewed
General Practice	General practitioner	4
Asylum Health Services	General practitioner	4
Community & Hospital Midwifery Services	Midwife	3
Sexual Health Services	Genito-urinary medicine doctor Health promotion specialist	4
Hospital services	Hospital doctor Dentist	6
Other allied health service	Community link workers	2

Of the 23 clinicians interviewed, 12 were women and 11 were men. They included three who self-identified as Chinese, two South Asians, two Black Africans and 14 White British.

The interviews and focus group discussions generated rich material within which three major thematic categories were identified (Tables 3, 4 and 5). One group of themes clustered around knowledge levels, as well as beliefs and attitudes of the three stakeholder groups. Another group pertained to the visibility of the issue and its priority relative to other competing demands. The third group were concerned with how services were configured and delivered. Below we summarise the main findings that were arrived at through the integrated analysis within and across the datasets. Selective quotations are provided in Table 3 to illustrate the type of data that was generated around particular themes.

**Table 3:** Poor knowledge, beliefs and perceptions of the population and the disease by the community, clinicians and commissioners

Themes	Definitions	Sample quotes
Knowledge of the disease among community members and patients	What the Chinese know about the disease and services (including myths and misunderstandings)	“[We] really know nothing about this (disease).” (Community focus group)
		“In China we heard that it’s curable. How come it becomes incurable in the UK?” (Community focus group)
		‘Are there services? Are they reliable?’ (Community focus group)
	What are the cultural norms, beliefs and expectations	“In China there are all sorts of stuff that doctors would prescribe to us to strengthen the immunity or detoxify the liver.” (Individual interviewee, person with hepatitis B)
		“What’s the point of taking all the blood tests, and (getting) no treatment?” (Individual interviewee, person with hepatitis B)
	What stigma exists about disease and persons with the disease, as well as associated services	“Chinese people believe HBV is easily transmitted through social contacts, so HBV carriers are often treated as a public nuisance, who are expected to inform people about his condition and keep their distance” (Community focus group)
		‘What if other people see me going into a sexual health clinic (for a hepatitis B test)? What will they think about me?’ (Individual interviewee)
Knowledge and attitudes of clinicians and commissioners	What clinicians and commissioners know of the disease and its management	“I reckon if you were to put down some hepatitis B results in front of any of us … I suspect we would probably have to go and have a little read on the internet or in the books.” (Clinician interviewee, General practitioner)
		“Because most of us trained more than ten years ago, there’s a perception that well there’s no point in treating hepatitis. So there’s something about educating the decision makers.” (Commissioner interviewee, regional public health consultant)
	What clinicians know about the Chinese and how they deal with it	“… we need to be careful about stereotyping people … there is a huge amount of in-group diversity.” (Clinician interviewee, hospital physician)
		“I’m hoping that there will be more ethnic training certainly in the primary care setting because as a primary care GP I think there’s desperately a lack of (training).” (Clinician interviewee, general practitioner)
	Perception of risk of infection in Chinese persons	“… perception that (the Chinese) are much easier because they don’t have any problems …” (Clinician)
		“I am not sure that any GP is going to have a sufficient population of Chinese to know that this is a major risk factor … If we make a bizarre comparison, if you see a Black patient you think of sickle cell. If you see a Chinese patient you don’t think about hepatitis B.” (Commissioner interviewee, regional public health consultant)

**Table 4:** Low visibility and relative priority of the disease and Chinese population

Themes	Definitions	Sample quotes
Visibility of the issue	How the community is perceived by clinicians and commissioners	“I think the culture within each group makes a difference as well. I think rightly or wrongly, the perception is that the Chinese immigrants tend to be more self-sufficient. The community takes on … new entrants and support them in a way that means they are not as visible to things like safeguarding or those other issues that flag them as a vulnerable group.” (Commissioner interviewee, local commissioning manager)
		“I think they probably do keep themselves to themselves… They are a quiet, self-contained group it seems to me…So because they haven’t upset people their needs aren’t immediately heard or seen.” (Clinician interviewee, general practitioner)
	How the community behaves that reduces its visibility	“(Eastern Europeans) … tend to demand more because they expect that … they ask a lot of things when they see the doctor. Whereas the Chinese population … if you are a doctor they do give you the respect. So that respect is there, which can be a barrier as well because then you know some of their hidden frustration may not surface...” (Clinician interviewee, Chinese-ethnicity general practitioner)
	Level of public discourse of the disease	“In China, HBV is frequently mentioned in everyday conversations, mass media, and internet. HBV is a hot and heavy topic, rumours of transmission together with tragic stories of suicidal HBV carriers encompassing people’s life.” (Community focus group)
		“In the UK (Hepatitis B) is hardly mentioned anywhere and has caught no public attention.” (Community focus group)
	Role for advocates	“There’s something about an advocate from within the community being a more successful voice for the change in terms of within the community … It’s something about a partnership between somebody who is willing to speak up for the community and somebody who’s willing to represent the community in the discussions to get a priority for it ...” (Commissioner interviewee, regional public health consultant)
		“Well I do believe we need the help from the (Chinese) population to push their own cause because other ethnic minority groups, when there’s a push from inside it’s more difficult to argue against I think.” (Clinician interviewee, general practitioner)
	Competing priorities	“They will make you take lots of medicine, and all the tests. I really haven’t got that much time.” (Community focus group)
		“If it’s only one or two cases in the city, frankly it’s not going to get the attention it deserves. If it’s hundreds or thousands, then it is more likely to. If it’s tens of thousands, it certainly will.” (Commissioner interviewee, local public health director)

**Table 5:** Structural barriers to health seeking and testing

Themes	Definitions	Sample quotes
Health system issues	Incentives, roles and responsibilities for providing hepatitis B-related services	“Then it comes to something like hepatitis screening. Good evidence for it in my opinion. Very logical to do. (But) there are all sorts of perverse incentives in the health system: so you are not paid (to test), there is no reward for being good at doing it … It costs you to do it, it takes time to do it, it’s hard to do it, it uses (hospital) referrals and actually is one of the areas where very often people are often over followed-up …” (Clinician interviewee, general practitioner)
	How one navigates the health system	“Maybe they have no understanding of the NHS system. Maybe they are new to this country. I mean if somebody said to me we want you to go to Bulgaria and sort yourself out a Hep B test, what the hell would I do? … that’s what we are trying to do here and that’s difficult.” (Commissioner interviewee, local commissioning manager)
	How accessible are health services	“Getting to see a doctor is harder than getting to meet an emperor.” (Community focus group)
		“They (the doctors) always sound as if you are making a mountain out of a molehill. They make you feel that you just don’t need to come. So I try not to bother my GP unless it is really serious...” (Community focus group)
	Whether services are tailored to the community	“I think the problem is that at the moment I don’t think services are particularly commissioned to take into account different ethnic groups or different areas of need.” (Commissioner interviewee, regional public health director)
		“Going out into communities is definitely the way forward for certain groups because … they wouldn’t (come in). Obviously language is a barrier and stigma. They don’t like to come into hospital.” (Commissioner interviewee, local commissioning manager)
		“It is really to get the understanding of what they would like, how they would like it, because anything we come up with might fall flat mightn’t it? That working with the communities is the key thing.” (Commissioner interviewee, regional public health consultant)
	What consultation and communication aids are present	“So if there’s a different language (involved) you know you definitely have to make sure that what you’ve said is being understood. And then there’s a lot more checking back as well when they answer.” (Clinician interviewee, general practitioner)
		“So although we tend to use phone interpreters, you can never be assured exactly what is being explained to (the patient).” (Clinician interviewee, general practitioner)

### Poor knowledge, awareness, beliefs and attitudes

There was poor knowledge of the disease amongst the Chinese participants interviewed, as well as among the clinicians and commissioners (Table 3). All three groups had knowledge gaps related to transmission routes, disease prevalence within the Chinese populations, and associated health complications. There was a lack of awareness of the asymptomatic nature of the infection among Chinese participants which contributed to delayed health-seeking and an erroneous underestimation of their personal risk of infection. Many were unaware of services available in the UK, and did not appreciate the need for long-term disease monitoring in those with chronic infections.

Poor understanding was evident among in-depth interview respondents who reported having the infection as well as those who participated in the focus group discussions. For example, some Chinese patients could not understand why tests were carried out on them but no treatment was provided (these patients presumably did not have active disease that required treatment and were probably being managed conservatively). This led to follow-up consultations being perceived to be *“a waste of time”.* Some Chinese participants expressed a mistrust of western medicine. Self-medication was commonly reported in the focus groups, including the use of traditional Chinese medicine and “*food therapy*” where certain herbal stews and drinks were consumed on health grounds *“to detoxify the liver”.* Importantly also, focus group discussions indicated that the belief that HBV is transmissible through shared food utensils was widespread. Community respondents reported difficulties in accessing reliable information in England and relied on Chinese language websites that were found to be inaccurate when examined by our clinical researchers and multi-lingual researcher.

Experience of stigma associated with the disease was evident among some, but not all, the community respondents, illustrating important heterogeneity within the group of people identifying as ‘Chinese’. Some participants voiced concerns of the repercussions of a HBV diagnosis such as the potential loss of employment and social exclusion. Some of this stemmed from their past experience of the disease’s stigma in their home countries – some focus group respondents and interview participants recounted how in their home countries infected persons were seen as a *“public nuisance”*. The Taiwanese group of Chinese associated the disease with *“moral deficit”*, caught by *“sleeping around”* or *“using needles”.* Some participants believed, perhaps erroneously, that they were not at risk as they led a *“stable life”*, did not use illicit drugs, and knew of no family members with the disease. Although individuals could freely access hepatitis B testing at genito-urinary medicine clinics, we found that these services also carried stigma and some Chinese participants worried about being seen at these clinics because of their association with sexually transmitted disease. Similarly, some respondents with the infection feared that attending hepatitis clinics could inadvertently result in the disclosure of their infection status if they were seen there. Due to the fears and shame of disclosure, patients also found it difficult to inform family members who were at risk. This situation was worsened by the fact that their families could be geographically dispersed.

Clinicians and commissioners interviewed also had poor awareness of the disease prevalence in this community. Many were unfamiliar with current testing protocols and national guidance (i.e. NICE guidance CG 165 and PH43) [24, 25]. This meant they did not always accurately perceive the risk of this disease in their Chinese patients and missed opportunities to offer testing.

### Low visibility of the issue

Another significant thematic group was the relatively low visibility of hepatitis B and, more generally, the health needs of the Chinese population in the UK (Table 4). In the focus group discussions participants noted that it was unusual to be discussing hepatitis B and that there was little social discourse about hepatitis B in the UK, both in the general public as well as within Chinese communities. Some clinicians also expressed the opinion that the Chinese used health services sparingly and tended not to prioritise their own health. Our community-level data supported this - new immigrants reported giving low priority to their own health (especially for asymptomatic conditions) in the first few years after arrival. Moreover, as they tended to be *“undemanding”*, busy clinicians were unlikely to delve into their health concerns fully or to proffer hepatitis B testing.

The lack of visibility also meant that both clinicians and commissioners were unaware of the high disease prevalence and attendant health needs in the Chinese. Our commissioner respondents described the Chinese as not being *“needy”* or a *“problem group”* and were perceived to have relatively good health compared to other ethnic groups. However, when pressed they admitted not having any evidence to support this view. Language barriers and the lack of strong advocates meant the needs of the Chinese were not articulated to commissioners. This situation was compared to that of some Eastern European groups who, it was observed, were the focus of considerably more vocal advocacy, such as from local politicians. As the Chinese were fairly dispersed nationally and present in small numbers in each locality, as one commissioner described, they were *“hidden in the statistics”*. In addition, other competing demands on limited commissioning resources meant their health needs were not seen as a commissioning priority. Consequently, respondents identified a need for greater advocacy for this population group in general, and this health issue in particular.

### Structural barriers to health seeking and testing

Barriers also exist and operate at different levels within the health system from the micro-level of the patient-provider interaction through to the macro-level of health service commissioning and policy making. At the patient-provider level, the health professionals identified issues such as the lack of language support and variable health literacy of interpreters (Table 5). At the service level, there was uncertainty as to whether HBV testing was funded within existing contractual arrangements. This meant clinicians were at times unsure whether they could offer it free of charge to patients. Similarly, at the policy level, commissioners reported there was a lack of clarity as to who was responsible for commissioning and providing HBV testing for this population. The provision of HBV-related care to this population came at a cost to healthcare providers and commissioners, both in terms of time and resources, and there were perverse disincentives within the system that deterred the provision of HBV testing. One clinician explained how they were not remunerated for carrying out routine HBV testing in primary care, and the identification of infected individuals implied added healthcare costs, as they were likely to be *“over-followed up”* (i.e. needed to be seen several times in hospital), at a time when cost-containment was a priority.

The needs of Chinese patients were also not always articulated and addressed due to language barriers. One prominent theme that emerged from all of the focus group discussions and many of the individual interviews with Chinese participants, across all age groups and educational status, was of their struggle to explain themselves to the health professionals. Similarly, the health professionals interviewed reported that consultations were more time consuming and that it was more difficult to check the Chinese patient’s understanding or concerns. Furthermore, the health professionals interviewed of Chinese ethnicity and those familiar with transcultural consultations observed that Chinese patients were unlikely to challenge their doctors’ decisions or to voice their concerns.

Several health professionals interviewed described their Chinese patients as uncomplaining, *“easy patients”* who rarely had any health problems. Many also had a poor understanding of Chinese culture or awareness of the considerable socioeconomic and cultural diversity within Chinese populations. Some practitioners did acknowledge their lack of knowledge and skills to adequately serve ethnic minority groups, and highlighted a need for further communication skills training.

The health system’s failure to accommodate patients with limited familiarity and poor English language skills was a significant barrier that affected the ability of Chinese patients to request tests and vaccinations for hepatitis B. Previous negative experiences of services in the UK exacerbated matters: Chinese respondents cited difficulties accessing services, rushed appointments, requests for tests being questioned by clinicians, a perception that doctors were withholding treatment due to budgetary constraints, and a lack of choice and control over the healthcare received. Some felt their concerns were trivialised by doctors, and several said that they tried *“not to bother the GP unless it is really serious.”*

Some clinicians acknowledged that services often did not address the needs of ethnic minority groups such as the Chinese and that there was a need for greater engagement with the community and adaptation of services to make them more patient-centered and responsive to local needs. We did encounter some local adaptations such as the use of community-based outreach services, *“one-stop”* clinics, flexible clinic times, and the use of language support services and Chinese-speaking community link workers. It was also suggested that reminders on the patients’ electronic health records could help prompt testing, and clinical protocols may help promote and standardise offers of testing. Commissioner input was highlighted as essential for creating an enabling environment for innovative tailored service approaches to be devised. The involvement of commissioners was also needed to ensure that these services would continue to be supported and funded.

## Discussion

In common with prior research [6, 26–28], we identified some individual and community-level factors that act as obstacles to appropriate testing and treatment for hepatitis B, including low awareness of the disease; inaccurate perceptions of personal infection risk; lack of familiarity navigating the health system; and stigma (particularly among some sub-groups). However, previously reported studies of interventions that focus narrowly on addressing knowledge and perceptions in minority Chinese populations have found weak, if any, effect on testing rates [29–31]. This suggests other determinants may operate and therefore different strategies may be required.

It was apparent from our study that clinician and commissioner knowledge and behaviours influenced their decisions with regards to whether Chinese patients were tested, or HBV-related services were provided, respectively. Stereotypes and misunderstandings about the population exist, reflecting a lack of cultural competence. Their lack of awareness of the population’s risk of infection means that opportunities to test are missed and hepatitis B-related services are unlikely to be commissioned. Moreover, how existing services were configured and delivered could adversely affect their accessibility and acceptability to the population. This situation is compounded by uncertainties with regards to who is responsible for commissioning and providing these services following the recent restructuring of health service design, purchasing and provision in England since 2012. Furthermore, the structures and responsibilities continue to change and regional variations exist.

Frontline clinicians may benefit from further education and training to develop cultural competency as well as communication skills to handle cross-cultural consultations. Other interventions that may help include the provision of services delivered in community-settings, services that operate more convenient flexible opening hours, and the use of community link workers. Multi-component interventions are likely to be needed but prior research has not to-date determined the efficacy of the individual components [12, 32]. Our study identified the following list of recommendations that may be beneficial (Table 6).

**Table 6:** Key recommendations

Community-level
▪ Improve knowledge about the disease, and awareness of the disease in at-risk groups ▪ Raise awareness of the asymptomatic nature of disease ▪ Tackle misperceptions ▪ Tackle stigma both at the individual and community level ▪ Provide health system navigators and clarify entitlements to health services ▪ Explore developing family support to influence testing and longer term compliance
Healthcare practitioners and services
▪ Improve healthcare practitioners’ knowledge of the disease, and raise their awareness of risk groups ▪ Develop cultural competency of staff. Be aware of in-group diversity ▪ Provide language support with health literate interpreters ▪ Train staff in the use of interpreters ▪ Make greater use of informational aids and tools such as patient alerts in electronic health records
Commissioners, Service Managers and Policymakers
▪ Ensure adequate resourcing for implementation of testing ▪ Identify advocate to champion the issue to local decision-makers. ▪ Engender leadership and ownership of the agenda ▪ Develop a compelling narrative of need and the economic case ▪ Clarify roles and responsibilities for commissioning hepatitis B services for migrants ▪ Develop joint collaborative commissioning where the responsibilities are split ▪ Encourage and empower local innovation ▪ Recognise community diversity and tailor responses to local needs and context ▪ Look to make services sustainable ▪ Increase accessibility of services - community-based/outreach services may help ▪ Explore collaboration with other agencies and the voluntary sector

Our findings echo previous work [33] that has highlighted the importance of commissioner engagement, especially when service adaptations are required in the context of resource constraints [34], in order to ensure that services are sustainable and adequately resourced. As the affected communities are diverse, interventions have to be tailored to local needs and context. This requires the delivery of services in different venues and ways, such as greater use of community outreach services, in order to improve their accessibility. However, interventions seeking to improve testing may still fail for a variety of reasons such as the short term nature of the evaluation of the interventions, costs that may be prohibitively expensive or deemed poor value for money, or accessibility issues. For services to be commissioned for the Chinese population, a compelling narrative of need and the economic case for commissioners is required. However, Chinese communities tend to be undemanding and hidden. Consequently, strong advocates to champion their needs to local decision-makers are needed. Greater visibility of the issue is crucial as it has to compete against other priorities that clinicians and commissioners have.

Stigma was a major theme in the data, operating at the individual, community and structural levels. The process of stigmatisation acts to keep people uninformed and stigma can also deter access to health services. Furthermore, stigma may potentially influence the priorities of health professionals and commissioners that could lead to certain health issues being addressed whilst others are ignored. The study findings also highlight an important tension between health literacy and illness representations among Chinese participants, clinicians and commissioners (i.e. what is known about hepatitis B compared to what an individual believes about hepatitis B). This tension needs to be better understood. Parallels can be drawn between HIV and hepatitis B in Chinese communities, and potentially useful insights could be extrapolated from research on HIV-related stigma [35, 36].

The cultural competence of institutions and healthcare workers needs to be further enhanced with an aim to achieve equal treatment despite cultural barriers. Accessibility issues to be addressed include the poor levels of awareness amongst clinicians and commissioners of the Chinese community’s health beliefs, behaviour and needs. Services have to ensure availability of effective language support with interpreters who are health literate, and staff must be adequately trained to make best use of interpreters. Greater use of informational aids and tools may also help [27].

In addition, it may be useful to consider the role of structural competency [37]. The clinical presentations of people from marginalized groups are shaped not only by cultural determinants, but also by multi-layered social, economic and political processes as well as structural inequities, biases and blind spots that produce inequalities in healthcare. Low levels of structural competency among commissioners and some clinicians could explain hepatitis B’s low visibility.

Furthermore, the UK previously had a targeted (selective vaccination for high risk newborns) rather than universal vaccination policy for hepatitis B as recommended by the World Health Organization, in view of the low prevalence of infection nationally. Consequently, vaccination programmes in the UK were unlikely to adequately protect existing or newly immigrating individuals with chronic HBV infection from high prevalence countries or their contacts, especially children, who may be at risk [1]. Routine immunisations for hepatitis B are to be introduced into the UK from the autumn of 2017 but will only cover newborns and not older children.

Feedback from our workshops suggested that existing health services should be encouraged to innovate and adopt different approaches to better engage with the target community. There is also a need to engage health commissioners in order to make this a priority for commissioning. A national profile for this issue could help raise this issue up local commissioning agendas. National drivers are especially influential in directing action locally when resources for commissioning are tight [18]. Crucially, clarity of organisational responsibilities for the commissioning and provision of hepatitis B services for migrant groups is required.

Whilst community education is recognized to be beneficial we need to explore further their informational needs and how information can be successfully delivered in practice. Further exploration of the role of family support to influence testing and longer term compliance may also be beneficial [38]. In the UK, the next steps needed are to design and test approaches to boost healthcare access and testing for hepatitis B in the migrant Chinese population. Those individuals who have a positive test will need to be subsequently followed up by the relevant clinical services, as well as informed that long term monitoring is important even in the absence of treatment.

As the participants were predominantly from South Yorkshire, the generalisability of the findings was a potential limitation which we sought to address through the national validation workshops. A key limitation was that we struggled to recruit male participants, and especially young men, as many were reluctant to do so or could not afford the time due to work demands. We were also unable to recruit Chinese individuals who were undocumented immigrants or sex workers. Whilst they may be less representative of the wider Chinese community, they are subgroups recognized to have a higher risk of hepatitis B infection [3]. Many of the barriers to hepatitis B testing and healthcare for these groups are probably similar to those faced by the wider Chinese community but there may be additional determinants not uncovered by this study. Finally, there are potential limitations with data generation when dealing with a sensitive topic – for example, participants may not wish to disclose their infection status or that of their family members. We were acutely aware of this and actively sought to conduct our community interviews sensitively.

## Conclusions

In a globalized world, public health systems often inadequately meet the needs of increasingly diverse multi-cultural populations that manifest as inequalities in health access. However, in the current global political context there is a real risk that this issue is conflated with arguments over national migration policy rather than as a public health issue. Currently, hepatitis B in the migrant Chinese in the UK is an invisible issue affecting an invisible population. Multi-modal solutions are required to address the various barriers that exist both in the community and the health system.

## Additional files

Additional file 1: Vignette and Focus Group Discussion Guide. Example of vignette used for focus group discussions, as well as discussion questions used in all the focus groups. (DOCX 20 kb)

Additional file 2: Community Key Informant Interview Guide. Interview schedule used for the individual key informant interviews with community participants. (DOCX 17 kb)

Additional file 3: Health Practitioner Interview Guide. Interview schedule used for the individual key informant interviews with health practitioners. (DOCX 18 kb)

Additional file 4: Policymakers-Health Commissioners Interview Guide. Interview schedule used for the individual key informant interviews with health commissioners, managers and policymakers. (DOCX 18 kb)

## Abbreviations

CG: Clinical Guidance
GP: General practitioner
HBV: Hepatitis B virus
NHS: National Health Service
NICE: The National Institute for Health and Care Excellence (UK)
PH43: Public Health Guidance No.43
