# Supplementary material for: Determinants of uptake of hepatitis B testing and healthcare access by migrant Chinese in the England: a qualitative study
Source: BMC Public Health. 2017 Sep 26;17:747. doi: 10.1186/s12889-017-4796-4 (PMC5615445; doi:10.1186/s12889-017-4796-4)
Supplement: Supplementary file 1 — Vignette and Focus Group Discussion Guide. Example of vignette used for focus group discussions, as well as discussion questions used in all the focus groups. (DOCX 20 kb) [file 12889_2017_4796_MOESM1_ESM.docx]

**VIGNETTE 1 FOR FOCUS GROUD DISCUSSION**

Jade is from China. She is 36 years old and has lived in the UK for 12 years. She is a chronic hepatitis B patient but she doesn’t attend any clinic because she generally feels well she doesn’t want to remind herself about this issue. She used to have a boyfriend but he left her after she told him about her HBV condition. Lucy is also from China; she is 34 years old and has lived in the UK for 10 years. Jade and Lucy are good friends. But Lucy doesn’t know that Jade is HBV positive. Lucy has recently separated with her husband and wants to rent a flat together with Jade. Jade welcomes her friend’s company but she is aware that Lucy has not had HBV vaccination. Jade doesn’t think that sharing a flat with her will increase Lucy’s risk of catching Hepatitis B, but she knows that Lucy thinks sharing food and cutleries with a HBV patient will largely increase the chance of catching the virus no mention living under the same roof. Jade doesn’t want to let Lucy know about her condition but tries to convince Lucy to have the test and vaccine. Lucy makes a phone call to her GP clinic. Her GP phones her back and refers Lucy to sexual health clinic. Lucy doesn’t understand why she should be referred there since she’s not worried about her sexual health, and she feels very uncomfortable of going to a clinic relating to ‘sexual health’. Lucy eventually decides to have the test and vaccine when she visits her parents in China next year. Lucy doesn’t understand why Jade is so keen on persuading her to have the test and vaccine. Jade doesn’t know if she should tell Lucy that she is HBV positive.

**FOCUS GROUP DISCUSSION GUIDE**

1. Should Jade tell Lucy about her HBV condition? Why? Why not?
2. What do you think are Jade’s concerns about disclosing her HBV condition?
3. Do you think the Chinese people in your community would find issues about HBV sensitive to discuss? Why do you think so?
4. Is Lucy right about the risk of HBV transmission? Is Jade right about the risk of HBV transmission?
5. How do the Chinese people in your community think HBV is transmitted?
6. What would you recommend Lucy and Jade to do before the vaccine and after the vaccine to prevent Lucy from catching the virus?
7. What should Jade do about her hepatitis B?
8. What do the Chinese people in your community think a person with hepatitis B could do to look after their health?
9. Should Jade have blood test regularly even if she feels completely well? Why and why not?
10. Do you agree with the actions of Lucy’s GP? Why? Why not?
11. Do you know anywhere else that Lucy could go to have the test and vaccine? Where?
12. Why do you think Lucy prefers to have her test and vaccine in China?
13. Is it common/popular for Chinese migrants go back to their home country for medical care? What do you think about it?
14. What do you think the NHS could do to prevent HBV spreading in the Chinese population in the UK?
15. Do the Chinese people in your community need more information and support about HBV and relevant medical care? How could this information and support be effectively delivered?
16. Do you have any other thoughts or comments you would like to add?
