# Supplementary material for: Determinants of uptake of hepatitis B testing and healthcare access by migrant Chinese in the England: a qualitative study
Source: BMC Public Health. 2017 Sep 26;17:747. doi: 10.1186/s12889-017-4796-4 (PMC5615445; doi:10.1186/s12889-017-4796-4)
Supplement: Supplementary file 2 — Community Key Informant Interview Guide. Interview schedule used for the individual key informant interviews with community participants. (DOCX 17 kb) [file 12889_2017_4796_MOESM2_ESM.docx]

**Key informant interview guide for community study**

The open-ended interviews explore in greater depth the issues identified from the FGDs. The interviews seek to understand:

1. Knowledge levels and understanding relating to hepatitis B (*knowledge levels’ connections to access to HBV care, the source of information* )
2. Attitudes towards the disease and (*risk perceptions’ connections to access to HBV care*)
3. Healthcare seeking behaviours (both in relation to NHS and non-NHS services)(*prompts and enablers*)
4. Attitudes and experiences of healthcare (*health ownership*)

(The black parts are original areas for exploration from CATH B proposal which have guided the FGDs. The italicised parts are the potential areas for further exploration in individual interviews)

The following are the questions asked in the open ended interview with key informants.

1. What do you think you need to know about hepatitis B?
2. What do you think you need to know about the healthcare services for hepatitis B?
3. Which sources of information do you use most to inform your health seeking?
4. How did you have your test/vaccination/check-up/treatment?
5. What made you have the test/vaccination/check-up/treatment?
6. How do you look after your health in general?
7. Do you have your preferred ways of keeping healthy? If yes, what are they? Why?
8. Under what circumstances will you seek help from health professionals?
9. Do you ever discuss with your doctor regarding your preference of treatment? Why yes, why not?
10. What do you do after seeing a doctor? Will you take the medicine? Why yes, why not?
11. Do you think immigrant life affect one’s health? If yes, in what way?
12. What can be done to support a HBV positive immigrant to access healthcare services in their early years of immigration?
