# Supplementary material for: Determinants of uptake of hepatitis B testing and healthcare access by migrant Chinese in the England: a qualitative study
Source: BMC Public Health. 2017 Sep 26;17:747. doi: 10.1186/s12889-017-4796-4 (PMC5615445; doi:10.1186/s12889-017-4796-4)
Supplement: Supplementary file 3 — Health Practitioner Interview Guide. Interview schedule used for the individual key informant interviews with health practitioners. (DOCX 18 kb) [file 12889_2017_4796_MOESM3_ESM.docx]

**Interview guide for practitioner study**

This guide helped to prompt practitioners and guide the interview but it not used as a questionnaire. Prompts were used if answers indicated the need for expanding or understanding. The questions also were adapted to the type of job and roles practitioners described. And some questions were added following initial interviews or when relevant information from other arms of the study became available.

Information sheet and consent form were sent in advance, and consent obtained immediately prior to the interview.

SAMPLE INTRODUCTION:

The information document explains this study aims to explore the issues of access to testing and healthcare services by Chinese people affected by hepatitis B. I am interested in your experience in this area as a front line worker/ key position.

The interview will last approximately 30 to 45 minutes; if any questions make you uncomfortable you don’t need to answer them and you are free to discontinue the interview at any point without giving a reason.

ROLE:

- Can you tell me what are your roles and what do they involve?
  - - Can you describe a typical day in your job? *
    - How does it differ from colleagues in similar roles?
    - Can you describe the responsibilities that each role involves? (i.e. clinical, educational, managerial, commissioning)

PATIENTS/INTERACTION/COMMUNICATION:

- What type of patients do you see? (Ethnicities, ages)*
- Do you see any Chinese patients?
  - - Are there any particular differences in the interaction with Chinese compared to other patients?
    - Are there any issues with communication, directly or through interpreters? Can you give examples?
- Can you remember a recent patient you have seen, can you describe the interaction?

HEPATITIS B

- What makes you think there is a risk for hepatitis B?
- Which particular groups you test regularly for hepatitis B, if any?
- How do you explain hepatitis B to a patient?
- What would be your response if a patient requests a test (for hepatitis B or other)? Can you explain why?

SUPPORT

- What support is there to help you carry out your responsibilities?
  - - Which resources do you have / use?
    - Can you think of any other support you may find useful?

BARRIERS TO INNOVATION / DEVELOPMENT:

- Are there any special provisions / innovation in your service?
- Can you tell me if you had experience in addressing service needs?
- How do you go about developing a service / new protocol / other?
- What do you think are ways to go about developing a service / new protocol / other?

EXTRA QUESTIONS**

- What is good/do you enjoy about your job and what do you do well?
- Is there anything else you think may be useful to know?
- Can you think of anything else that can act as barriers for patients (Chinese or other) to access hepatitis B testing or healthcare?

** Icebreaker questions, soft introductions
** Concluding questions / prompts for ideas that may surface at the end*
