# Supplementary material for: Determinants of uptake of hepatitis B testing and healthcare access by migrant Chinese in the England: a qualitative study
Source: BMC Public Health. 2017 Sep 26;17:747. doi: 10.1186/s12889-017-4796-4 (PMC5615445; doi:10.1186/s12889-017-4796-4)
Supplement: Supplementary file 4 — Policymakers-Health Commissioners Interview Guide. Interview schedule used for the individual key informant interviews with health commissioners, managers and policymakers. (DOCX 18 kb) [file 12889_2017_4796_MOESM4_ESM.docx]

## Commissioners and Policymakers Interview Guide

ROLES & RESPONSIBILITIES

1. What role do you have currently (or have you had in the past) with regards to either Hepatitis B or services for the Chinese ethnic group in the UK?
   1. What is the role of commissioners with regards to either Hepatitis B or services for the Chinese ethnic group in the UK?

PROCESS

1. What is the process involved for getting services commissioned for Hep B for this ethnic group?

BARRIERS AND FACILITATORS

1. What is necessary/required for this to happen?
   1. In your experience what do you think are the barriers to commissioning Hepatitis B services for this ethnic group?
   2. From your experience, how much of an issue is Hepatitis B in this ethnic group in the UK?
      1. Why is that?
      2. Are there any special considerations required for this ethnic group?
   3. Is this a priority issue for commissioners?
      1. What’s stopping it being a priority?
      2. How do we make it a priority?
   4. What are the facilitators to commissioning Hepatitis B services for this ethnic group?

ROLE OF GUIDANCE

1. Are you familiar with the NICE Guidance for Hepatitis B?
   1. Is this guidance being implemented? How much of this guidance is being implemented?
   2. What are the barriers and facilitators to implementation of this guidance?
   3. What changes would we need to make?
2. If we were wanting to improve service provision to increase Hepatitis B testing in this target ethnic group, what advice would you give other commissioners/policymakers
